# The Impact of Surfactant Protein‐D Gene Polymorphism on COVID‐19 Clinical Outcomes

**DOI:** 10.1002/iid3.70410

**Published:** 2026-05-15

**Authors:** Nadia Nasirzadeh Kolsari, Azarakhsh Azaran, Roya Pirmoradi, Maryam Moradi, Roohangiz Nashibi, Shahram Jalilian

**Affiliations:** ^1^ Infectious and Tropical Diseases Research Center, Health Research Institute Ahvaz Jundishapur University of Medical Sciences Ahvaz Iran; ^2^ Department of Medical Virology, School of Medicine Ahvaz Jundishapur University of Medical Sciences Ahvaz Iran; ^3^ Department of Biostatistics and Epidemiology, School of Public Health Ahvaz Jundishapur University of Medical Sciences Ahvaz Iran

**Keywords:** COVID‐19, polymorphism, pulmonary surfactant‐associated protein D, SARS‐CoV‐2, single nucleotide

## Abstract

**Background:**

COVID‐19, caused by the severe acute respiratory syndrome coronavirus 2 (SARS‐CoV‐2), presents a broad spectrum of clinical manifestations, ranging from asymptomatic cases to severe, life‐threatening respiratory complications. Pulmonary surfactant‐associated protein D (SP‐D) is a critical component of pulmonary immune defense. The objective of this study was to investigate the association between a specific single‐nucleotide polymorphism (SNP) in the SP‐D gene, designated as rs721917 (C/T Met31Thr), and its potential impact on susceptibility to and severity of COVID‐19.

**Methods:**

This retrospective case–control study enrolled 135 participants, including 111 confirmed COVID‐19 patients and 24 asymptomatic or presymptomatic individuals, who were classified into five subgroups. Addressing a significant research gap, we identified the C/T polymorphism (rs721917, T > C) of the SP‐D gene within the Iranian population using the tetra‐amplification refractory mutation system polymerase chain reaction (T‐ARMS PCR) method.

**Results:**

Statistical analysis showed a significant association between the SP‐D rs721917 TT genotype and T allele with increased COVID‐19 severity and hospitalization risk. The TT genotype was more frequent in ICU and CCU‐admitted patients compared to the CC genotype (*p* < 0.05) and was linked to higher hospitalization rates overall. However, no significant correlation was found with mortality rates or patients' age.

**Conclusion:**

These findings suggest that variations at the rs721917 locus within the SFTPD gene may provide valuable insights into the heterogeneity of COVID‐19 outcomes across different populations, thereby offering potential for enhanced diagnostic and prognostic strategies.

## Summary

- SP‐D polymorphism and outcome of COVID‐19 symptoms.

## 1 Introduction

In December 2019, a novel and highly contagious virus emerged in Wuhan, China, eliciting widespread concern on a global scale. The outbreak, designated COVID‐19, is attributed to the severe acute respiratory syndrome coronavirus 2 (SARS‐CoV‐2). The rapid dissemination of SARS‐CoV‐2 across international borders resulted in a substantial number of infections and profound socio‐economic disruptions, culminating in a global crisis [1]. The World Health Organization's declaration of the virus as a pandemic on March 11, 2020, presented an unprecedented challenge to global public health, marking a significant milestone in modern medicine and global health [2]. The crystal structure of the SARS‐CoV‐2 spike protein reveals that the receptor‐binding domain interacts with a cellular receptor known as angiotensin‐converting enzyme 2 (ACE2), forming a complex [3]. The ACE2 receptor is predominantly located in alveolar epithelial type II (AE II) cells within the lungs and serves as the primary target for SARS‐CoV‐2 [4]. Furthermore, AE II cells are primarily responsible for synthesizing and secreting pulmonary surfactant, a lipoprotein substance that plays a critical role in reducing surface tension within the alveoli [3].

SARS‐CoV‐2 can affect both the upper and lower respiratory systems, presenting a spectrum of clinical manifestations that ranges from asymptomatic cases to severe conditions. Research indicates that advanced age, along with preexisting medical conditions or comorbidities, is associated with increased disease severity [5]. The accurate and timely identification of COVID‐19 is crucial for enhancing the management and treatment of this potentially life‐threatening disease [6]. Emerging evidence underscores the significance of single‐nucleotide polymorphisms (SNPs) in relation to fatality rates and disease severity [1]. Recent studies have suggested a potential correlation between surfactant deficiency and the onset of acute respiratory distress syndrome (ARDS) in patients with COVID‐19 [7, 8]. Surfactant protein D (SP‐D), a member of the collectin protein family, is synthesized by AE II cells and club cells within the lungs. SP‐D consists of homotrimeric subunits, each containing an N‐terminal domain with conserved cysteines essential for subunit cross‐linking, followed by a collagen‐like domain, an α‐helical neck, and a C‐type lectin carbohydrate recognition domain (CRD). SP‐D typically assembles into cruciform structures composed of four trimeric subunits, although it can also form higher‐order oligomers under physiological conditions [9]. This protein plays a pivotal role in the innate immune response, mediating allergic reactions and mitigating lung inflammation [10, 11]. SP‐D binds to microorganisms, thereby facilitating their clearance through opsonization and phagocytosis. Additionally, it effectively regulates the progression of various viral infections and serves as a significant barrier against enveloped viruses [3, 12]. Human amniotic fluid is a significant source of SP‐D. Studies using SP‐D derived from this fluid have shown that its ability to interact with bacteria and viruses depends on the increased avidity provided by multimer formation. Interestingly, trimeric SP‐D—selected based on size rather than affinity—demonstrated stronger binding to lipopolysaccharides (LPS) than multimeric forms, indicating that different oligomeric states may have distinct functional characteristics [13]. COVID‐19 has been shown to impair surfactant production by type II alveolar cells, with proteins such as SP‐A and SP‐D emerging as potential biomarkers for pulmonary injury [14]. SNPs and genetic variations are critical in the pathogenesis of the disease, influencing infection spread and the development of effective treatments. Furthermore, SP‐D modulates microbial adherence to the host, immune responses, susceptibility to diseases, and the overall severity of COVID‐19 [15].

SP‐D enhances pathogen clearance by cooperating with alveolar macrophages and promoting phagocytosis through opsonization. The rs721917 (Met31Thr) polymorphism in SP‐D affects the protein's oligomerization and function. The Met31 variant tends to form multimeric structures that have a higher affinity for pathogens, while the Thr31 variant primarily produces monomers that preferentially bind to LPS. These structural differences may influence the efficiency of the immune response, although the full biological impact is still not fully understood [16, 17]. A specific SNP, designated rs721917, has been identified within the coding sequence of the SP‐D gene (SFTPD). This SNP, also referred to as Met31Thr, results in the substitution of methionine with threonine at position 31 in the mature SP‐D protein, thereby exerting a significant impact on its function [11]. Shakoori et al. reported a notable association between the TT genotype of the rs721917 polymorphism and increased susceptibility to chronic obstructive pulmonary disease (COPD). Furthermore, they found that the presence of the C allele in this SNP was correlated with decreased serum levels of SP‐D [18]. The rs721917 polymorphism is also associated with various disorders, including severe respiratory syncytial virus infection [19], allergic rhinitis [20], coronary stenosis [21], type II diabetes [22], lung cancer [23], rheumatoid arthritis [24], tuberculosis [25], and atherosclerosis [26]. These associations suggest a role for this polymorphism in the development of these diseases and underscore the influence of genetic mutations on disease susceptibility and SP‐D concentrations.

Previous studies have demonstrated that patients infected with COVID‐19 exhibit elevated levels of serum SP‐D when compared to healthy individuals [27]. A recent meta‐analysis revealed a significant association between elevated SP‐D levels and severe outcomes in COVID‐19, thereby highlighting its potential as a prognostic biomarker. This finding emphasizes the role of SP‐D in disease progression and its utility in identifying individuals at high risk [28]. Furthermore, increased serum SP‐D levels have been correlated with severe cases of COVID‐19, in conjunction with specific laboratory parameters [29]. Currently, there is a paucity of data investigating the relationship between variations in the SFTPD gene and the severity of COVID‐19. Consequently, the aim of the present study is to ascertain whether there exists an association between the SFTPD gene SNP rs721917 (NC_000010.11:g.79946568A > G or NG_042218.1:g.7538T > C at position chr10:79946568, GRCh38.p14) and the various stages of COVID‐19 among Iranian populations. The findings of this research will provide valuable insights for targeted risk assessments and therapeutic interventions.

## 2 Materials and Methods

### 2.1 Study Design and Subject Criteria

This retrospective case–control study comprised a total of 135 participants and received approval from the Ethics Committee of Jundishapur University of Medical Sciences, Ahvaz, Iran (IR.AJUMS.REC.1401.503). Between February and May 2022, 111 COVID‐19‐positive patients were admitted to Razi Hospital in Ahvaz, Iran. These patients were identified based on clinical symptoms and confirmed through positive results from nasopharyngeal and/or oropharyngeal swabs utilizing Qualitative PCR methodology. They were hospitalized primarily due to symptoms such as fever, dyspnea, or other respiratory manifestations. The control group consisted of 24 asymptomatic or presymptomatic individuals who had tested positive for COVID‐19 via PCR but exhibited no symptoms of the disease or respiratory conditions, and no underlying health issues. Their positive test results were obtained from the Qualitative PCR test conducted at Allameh Karami Hospital. This control group was matched with the case group based on demographic and health‐related factors, including age, gender, weight, and the absence of smoking history or relevant medical conditions.

### 2.2 Definitions and Diagnosis

COVID‐19 patients were categorized into five groups (see Table 1). Asymptomatic or presymptomatic infection pertains to individuals who tested positive for SARS‐CoV‐2 through virologic assessments, including nucleic acid amplification tests (NAATs) and antigen tests, yet exhibited no symptoms of COVID‐19. Mild illness was operationally defined as the condition of outpatients exhibiting mild symptoms, possessing normal radiological findings, maintaining an oxygen saturation (SpO_2_) greater than 94% at sea level as measured by pulse oximetry, and demonstrating a respiratory rate < 24 breaths per min, without dyspnea or shortness of breath. Moderate illness encompassed hospitalized patients presenting with fever, pneumonia, and radiological evidence of pneumonia as observed on chest computed tomography (CT) scans. These patients were required to maintain SpO_2_ levels of 94% or higher. Severe pneumonia was defined by the presence of respiratory distress, an SpO_2_ < 94% at sea level, a PaO_2_/FiO_2_ ratio < 300 mmHg, respiratory frequency > 30 breaths per min, or pulmonary infiltrates affecting more than 50% of the total lung area. Critical cases had SpO_2_ < 90% and required mechanical ventilation for respiratory failure, with the potential for septicemia and multiple organ failure [30]. Admission to the intensive care unit (ICU) was necessary due to respiratory failure. Patients in the mild‐to‐moderate stage were categorized as nonsevere cases, while severe‐to‐critical cases were classified as severe cases.

**Table 1:** Participant characteristics: Demographic profiles and clinical/paraclinical characteristics.

Characteristics	Covid‐19 severity (median, IQR) count (expected count)				Asymptomatic or presymptomatic patients (median, IQR)	*p* value, Kruskal–Wallis *H* test
	Mild	Moderate	Severe	Critical		
Number (mean ± SD)	15 (11.1)	42 (32.6)	37 (27.4)	17 (12.6)	24 (17.8)	
Age(year)	(Median = 55)	(Median = 59.50)	(Median = 70)	(Median = 58)	(Median = 38.50)	< 0.05
(Median, IQR)	(IQR = 19)	(IQR = 18.25)	(IQR = 20)	(IQR = 19.5)	(IQR = 18.75)	
Duration of hospital stay (days)	(Median = 0)	(Median = 2.5)	(Median = 6)	(Median = 5)	—	< 0.05
(Median, IQR)	(IQR = 0)	(IQR = 5)	(IQR = 5)	(IQR = 10)		*P* value chi‐square test
BMI (Normal)	3 (4.3)	12 (12.1)	11 (10.7)	6 (4.9)	7 (6.9)	0.889
Overweight	9 (8.7)	24 (24.3)	21 (21.4)	8 (9.8)	16 (13.9)	0.296
Obese	3 (2)	6 (5.6)	5 (4.9)	3 (2.3)	1 (3.2)	
Male	5 (6.4)	21 (18)	15 (15.9)	8 (7.3)	9 (10.3)	0.752
Female	10 (8.6)	21 (24)	22 (21.1)	9 (9.7)	15 (13.7)	
Smoking	3 (1.9)	7 (5.3)	4 (4.7)	3 (2.1)	—	0.254
Fever	8 (7.4)	23 (20.8)	26 (18.4)	10 (8.4)	—	< 0.05
Dry cough	12 (10.3)	32 (28.9)	34 (25.5)	15 (11.7)	—	< 0.05
Productive cough	1 (2)	5 (5.6)	9 (4.9)	3 (2.3)	—	0.075
Myalgia/fatigue	2 (3.3)	12 (9.3)	12 (8.2)	4 (3.8)	—	< 0.05
Dyspnea	6 (8.1)	18 (22.7)	32 (20)	17 (9.2)	—	< 0.05
Tachypnea	1 (2.6)	4 (7.2)	9 (6.3)	9 (2.9)	—	< 0.05
Nausea or vomiting	1 (0.8)	3 (2.2)	3 (1.9)	0 (0.9)	—	0.512
Loss of taste‐smell	4 (1)	3 (2.8)	1 (2.5)	1 (1.1)	—	< 0.05
Hypertension	3 (4.7)	20 (13.1)	15 (11.5)	4 (5.3)	—	< 0.05
Diabetes mellitus	3 (3.8)	13 (10.6)	10 (9.3)	8 (4.3)	—	< 0.05
Cancer	0 (0.9)	4 (2.7)	3 (2.4)	2 (1.2)	—	0.421
Corticosteroids	0 (1.3)	1 (3.7)	7 (3.3)	4 (1.5)	—	< 0.05
Remdesivir	1 (7.3)	14 (20.5)	35 (18.1)	16 (8.3)	—	< 0.05
Hydroxychloroquine/chloroquine	0 (2.4)	5 (6.8)	9 (6)	8 (2.8)	—	< 0.05
Tocilizumab	0 (0.7)	0 (1.9)	3 (1.6)	3 (0.8)	—	< 0.05
Outpatiant	15 (3.3)	15 (9.3)	0 (8.2)	0 (3.8)	—	< 0.05
Infectious ward	0 (4.3)	20 (12.1)	16 (10.7)	3 (4.9)	—	< 0.05
Cardiac care unit	0 (2.2)	5 (6.1)	11 (5.3)	0 (2.5)	—	< 0.05
Intensive care unit	0 (3.5)	2 (9.8)	10 (8.7)	14 (4)	—	< 0.05
						< 0.05
Survived cases	15 (13.6)	42 (38.2)	32 (33.7)	12 (15.5)	—	< 0.05
Mortality cases	0 (1.4)	0 (3.8)	5 (3.3)	5 (1.5)	—	< 0.05

*Note:* BMI was calculated by dividing weight in kilograms by height in meters squared. Statistical significance was considered at a significance level: A statistically significant difference was observed among the patient groups, as determined by the Kruskal–Wallis *H* test and the chi‐square test (*p* < 0.05).

Abbreviations: BMI, body mass index; COVID‐19, coronavirus 2019.

### 2.3 Data Collection

Patient assessments involved the consideration of multiple factors, including the severity of the condition, medical history, and relevant comorbidities (e.g., diabetes, hypertension, and recent surgical interventions). Evaluative measures encompassed clinical presentations, chest CT scans, and laboratory tests, such as fasting blood glucose levels, complete blood count (CBC), and C‐reactive protein levels. Consultation with specialists was sought for various conditions, including diabetes, hypertension, human immunodeficiency virus (HIV), and heart failure. Furthermore, daily updates on biochemical, hematological, coagulation, and arterial blood gas parameters were performed upon admission and are reported in this study.

### 2.4 Genomic DNA Extraction Protocol

To isolate genomic DNA, 200 μL of peripheral blood mononuclear cells obtained from each participant were utilized. The Sinacolon DNA Extraction Kit DNP, IRAN, was employed in accordance with the manufacturer's protocol. The quality of the isolated genomic DNA was evaluated using NanoDrop spectrophotometry (ND‐1000, Thermo Fisher Scientific). To ensure long‐term stability and suitability for subsequent analyses, the isolated genomic DNA was stored at −20°C.

### 2.5 Amplification of the Targeted Segment by Tetra‐Arms‐PCR

Gene‐specific primers were designed utilizing the SNPgen SNP Genotyping Assay Designer portal. Subsequently, the Tetra‐primer amplification refractory mutation system‐polymerase chain reaction (Tetra‐Arms‐PCR) was conducted to amplify the target gene. Verification of the distinct DNA band was accomplished through electrophoresis on a 2% agarose gel.

### 2.6 Genotyping to Detect SNP rs721917

The Tetra‐ARMS‐PCR technique facilitates the simultaneous amplification of both wild‐type and mutant alleles, in addition to a control fragment, within a single reaction. This methodology employs two standard outer primers targeting the locus of interest, which generate a non‐allele‐specific control fragment. Furthermore, two inner primers, each specific to an allele and oriented oppositely, are utilized. The specificity of these inner primers for either the variant or wild‐type allele is achieved through the intentional incorporation of a mismatched nucleotide at their 3′ ends. The resultant amplicons exhibit distinguishable lengths, thereby allowing for facile differentiation via standard gel electrophoresis. This length variation arises from the asymmetric positioning of the mutation relative to the standard primers. Tetra‐ARMS‐PCR offers the advantage of consistently yielding at least one allele‐specific fragment alongside a control fragment, thereby providing an internal control to identify false negatives and mitigate amplification failures. In the genotyping of the SFTPD polymorphism C/T, rs721917 T > C, the tetra‐amplification refractory mutation system PCR method was utilized. The designated primers for outer amplification were F1 (5′‐CAACTGCTAACTGCTGGCACA‐3′) and R1 (5′‐AGCTGGGTAACTCATGCTCT‐3′). For inner amplification, primers F2 (5′‐AGGGTGCAAGCACTGGTCA‐3′) and R2 (5′‐ACCTACTCCCACAGAACCAC‐3′) were employed.

To detect SNPs, we amplified DNA under specific conditions. The reaction comprised 4 µL of DNA (approximately 100 ng/µL), 1 µL of primers, 12.5 µl of Taq 2X Master Mix, and 4.5 µL of distilled water. The protocol commenced with an initial temperature of 110°C, followed by denaturation at 95°C for 5 min. Subsequently, we executed 40 cycles consisting of denaturation at 94°C for 30 s, annealing at 58°C for 30 s, and extension at 72°C for 1 min. A final extension at 72°C for 5 min concluded the thermal cycling. We evaluated the PCR products and identified SNPs via gel electrophoresis utilizing a 2% agarose gel stained with DNA‐safe dye. The visualization of PCR results was achieved using a Gel Imaging Instrument (EZ, Bio‐Rad). SNPs denote genetic variations occurring at specific nucleotide positions, involving adenine, thymine, cytosine, or guanine. When conducting practical T‐ARMS PCR for SNP detection, it is vital to consider factors such as DNA extraction, annealing temperature, PCR cycles, reagent selection, and primer concentrations, with the annealing temperature being particularly critical.

We performed two PCR reactions employing specific primer pairs to amplify the T allele (316 bp) and the C allele (441 bp). The PCR DNA products were purified using the Zymoclean Gel DNA Recovery Kit and subsequently sent to the genetic laboratory at Shahid Chamran University for sequencing. The sequencing procedure was executed using an Applied Biosystems 3500 Genetic Analyzer.

### 2.7 Statistical Analysis

The sample size was determined using G*Power software, applying a significance level of 0.05 and a power level of 0.80. Data analysis was conducted using SPSS version 26.0 for Windows. Normality of continuous variables was assessed using the Kolmogorov–Smirnov test. Based on these results, quantitative data were expressed as median and interquartile range (IQR) for non‐normally distributed variables (including age and laboratory parameters). Qualitative results were reported in terms of frequencies and percentages. Categorical variables were compared using the chi‐square test for independence. Observed and expected values are reported for these variables. Continuous variables (age and laboratory parameters) that were non‐normally distributed were compared across the independent groups using the Kruskal–Wallis *H* test. To assess the association between genotype/allele frequencies and the severity of COVID‐19 and the frequency of the outcome across genotype groups, the Chi‐square used for independence. Specifically, the chi‐square test was applied to compare the observed genotype frequencies (TT, CT, and CC) in each disease severity group against the observed frequencies in the asymptomatic group.

For overall comparisons of key clinical parameters across the three independent groups (e.g., Mild, Moderate, and Severe/Critical), the Kruskal–Wallis test was employed. When the Kruskal–Wallis test showed a statistically significant overall difference (*p* < 0.05), the nonparametric Dunn's post hoc test was subsequently performed for pairwise comparisons (e.g., Severe vs. Non‐Severe). The adjusted *p* values (using a method such as Bonferroni correction) for these specific pairwise comparisons were presented.

## 3 Result

### 3.1 Demographic, Clinical Features, and Laboratory Data

The patients in this study were classified according to sex and age demographics. A total of 135 participants were included, of whom 111 were diagnosed with COVID‐19. Among these, there were 15 cases categorized as having mild illness, 42 cases of moderate illness, 37 cases of severe illness, and 17 cases classified as critical illness. Additionally, 24 patients were identified as asymptomatic or presymptomatic, all of whom tested positive for COVID‐19 via PCR. The correlation among various clinical symptoms associated with COVID‐19, including fever, dry cough, myalgia, shortness of breath, and loss of taste and smell, was found to be statistically significant at different stages of the disease (*p* < 0.05). Among the patients undergoing treatment, 7.4% required intubation, and all of these patients were classified within the severe disease group. The study identified a total of nine cases of cancer, two cases of human immunodeficiency virus infection (HIV), two cases of hepatitis C virus infection (HCV), one case of Epstein–Barr virus (EBV), and three cases of Mycobacterium tuberculosis infection (TB). However, no significant correlation was observed between these comorbid conditions and disease severity (*p* > 0.05). Notably, no patients exhibited all three comorbidities simultaneously, which may be attributed to the limited sample size of the study population. A comprehensive breakdown of the clinical signs, demographic characteristics, and symptoms of COVID‐19 is provided in Table 1, which also reports the observed counts and expected counts, along with the corresponding *p* values to evaluate statistical significance.

In cases of severe COVID‐19, there was a notable elevation in leukocyte count, D‐dimer levels, blood inflammation parameters, and hospitalization duration when compared to non‐severe cases (*p* < 0.05). Furthermore, there were noteworthy discrepancies in various biochemical parameters between severe and non‐severe cases, including erythrocyte sedimentation rate (ESR), aspartate aminotransferase (AST), Alanine aminotransferase (ALT), bilirubin, D‐dimer, creatinine, and procalcitonin (Table 2).

**Table 2:** Laboratory findings of COVID‐19 patients in different disease stages.

Severity *n* (%) (mean ± SD)						
Parameter	Total patients	Mild	Moderate	Severe	Critical	*p* value, Kruskal–Wallis test
Number of patients	111 (82.2)	15 (11.1)	42 (32.6)	37 (27.4)	17 (12.6)	
Blood markers						
WBC count (×10^3^/L)	9.70 ± 5.45	7.15 ± 3.05	7.56 ± 3.17	12.18 ± 7.18	11.86 ± 4.17	< 0.05
RBC count (×10^6^/L)	4.15 ± 0.74	4.50 ± 0. 44621	4.19 ± 0.72289	4.17 ± 0.78094	3.71 ± 0.75953	< 0.05
Lymphocytes count (×10^3^/L)	23.53 ± 15.12	40.46 ± 15.71	25.41 ± 11.93	19.27 ± 14.71	13.25 ± 8.51	< 0.05
Neutrophil count (×10^3^/L)	66.49 ± 21.40	59.23 ± 18.69	67.44 ± 15.01	73.04 ± 19.82	56.25 ± 33.46	< 0.05
HCT (%, L/L)	35.00 ± 7.08	38.007 ± 6.34	35.360 ± 6.18	34.99 ± 7.75	31.48 ± 7.39	0.07
Plt count (×10^3^/L)	274.72 ± 115.08	287.80 ± 91.352	270.48 ± 94.739	261.30 ± 129.52	302.88 ± 146.748	0.392
Hb (g/dL)	11.39 ± 2.16	12.16 ± 1.68	11.48 ± 1.97	11.55 ± 2.34	10.16 ± 2.25	0.072
Iron (mg/dL)	81.08 ± 11.86	80.80 ± 7.702	79.02 ± 13.46	81.95 ± 12.86	84.53 ± 7.26	0.558
Metabolic						
Sodium (mg/dL)	137.42 ± 4.16	137.13 ± 3.701	138.07 ± 3.97	136.03 ± 3.86	139.12 ± 4.93	0.074
Potassium (mg/dL)	7.80 ± 37.66	30.64 ± 102.45	4.236 ± 0.5716	4.26 ± 0.5452	4.16 ± 0.5291	0.962
Calcium (mg/dL)	8.55 ± 0.89	8.107 ± 2.0509	8.68 ± 0.5426	8.50 ± 0.4576	8.76 ± 0.5612	0.438
Phosphorus (mg/dL)	3.20 ± 1.02	3.080 ± 0.9420	3.26 ± 1.24	2.99 ± 0.6304	3.60 ± 1.13	0.129
Magnesium (mg/dL)	2.01 ± 0.23	1.96 ± 0.1957	1.98 ± 0.1612	2.09 ± 0.3087	1.95 ± 0.1772	0.265
BS (mg/dL)	140.73 ± 60.01	126.20 ± 50.43	123.95 ± 44.22	160.46 ± 68.041	152.06 ± 71.64	< 0.05
Inflamations markers						
CRP (mg/dL)	54.71 ± 61.96	11.27 ± 9.45	33.69 ± 40.01	67.77 ± 65.80	116.58 ± 71.26	< 0.05
IL‐6 (pg/mL)	6.70 ± 8.07	3.28 ± 0.85957	3.86 ± 0.95530	7.19 ± 6.90	15.70 ± 14.80	< 0.05
ESR (mL/heartbeat)	35.43 ± 28.28	16.98 ± 9.57	23.11 ± 14.94	50.81 ± 33.98	48.70 ± 28.25	< 0.05
D‐dimer (ng/mL)	408.96 ± 1186.69	153.20 ± 46.33	196.81 ± 216.80	289.94 ± 243.47	1417.84 ± 2851.72	< 0.05
Biochemical markers						
Direct bilirubin (mg/dL)	0.22 ± 0.05	0.213 ± 0.0352	0.214 ± 0.0521	0.222 ± 0.0534	0.241 ± 0.0795	0.321
Total bilirubin (mg/dL)	1.09 ± 1.64	0.727 ± 0.1668	1.34 ± 2.64	0.986 ± 0.2800	1.01 ± 0.2628	< 0.05
AST (U/L)	38.51 ± 34.08	29.93 ± 8.64	35.79 ± 40.27	41.81 ± 37.71	45.65 ± 19.87	< 0.05
ALT (U/L)	34.17 ± 58.58	22.80 ± 9.39	38.17 ± 90.95	33.57 ± 28.70	35.65 ± 17.24	0.137
LDH (U/L)	548.21 ± 239.28	354.47 ± 46.51	501.19 ± 162.70	610.59 ± 253.221	699.53 ± 324.54	< 0.05
Albumin (g/dL)	4.06 ± 0.68	3.97 ± 0.4574	4.07 ± 0.6510	4.17 ± 0.7162	3.90 ± 0.8525	0.606
Procalcitonin (ng/mL)	0.23 ± 0.13	0.20600 ± 0.086833	0.24833 ± 0.152698	0.26630 ± 0.152104	0.17959 ± 0.056606	0.288
Kidney function						
Creatinine (mg/dL)	1.30 ± 1.41	0.813 ± 0.2532	1.41 ± 2.09	1.25 ± 0.8170	1.54 ± 0.7977	< 0.05
BUN (mg/dL)	24.05 ± 17.15	15.67 ± 5.21	22.48 ± 16.22	25.89 ± 19.69	31.29 ± 17.81	< 0.05
Uric acid (mg/dL)	6.60 ± 1.23	5.14 ± 1.28	6.47 ± 1.04	6.86 ± 0.9869	7.64 ± 0.8639	< 0.05
Blood coagulation markers						
INR (s)	1.15 ± 0.28	1.06 ± 0.0910	1.09 ± 0.1574	1.25 ± 0.4388	1.18 ± 0.1667	< 0.05
PT (s)	12.92 ± 1.63	12.20 ± 0.4140	12.76 ± 1.29	13.32 ± 2.31	13.11 ± 0.9926	< 0.05
PTT (s)	37.44 ± 10.90	35.73 ± 2.604	37.13 ± 13.61	39.89 ± 11.28	34.41 ± 4.87	0.269
Respiratory markers						
pH	7.40 ± 0.09	7.40 ± 0.043176	7.38 ± 0.086115	7.39 ± 0.097588	7.44 ± 0.108806	0.06
PCO_2_ (mm/Hg)	42.13 ± 7.20	42.71 ± 4.99	42.09 ± 7.67	42.04 ± 7.59	41.94 ± 7.32	0.951
PO_2_ (mm/Hg)	56.14 ± 26.49	60.92 ± 12.96	63.80 ± 29.70	47.84 ± 28.25	51.03 ± 16.08	< 0.05

*Note:* Kruskal–Wallis test, *p* < 0.05 was considered statistically significant.

Abbreviations: ALT, alanine aminotransferase; AST, aspartate aminotransferase; BS, blood sugar; BUN, blood urea nitrogen; CRP, C‐reactive protein; ESR, erythrocyte sedimentation rate; Hb, hemoglobin; HCT, hematocrit; IL‐6, interleukin‐6; INR, international normalized ratio; LDH, lactate dehydrogenase; PCO_2_, partial pressure of carbon dioxide; PLT, platelets; PO_2_, partial pressure of oxygen; PT, prothrombin time; PTT, partial thromboplastin time; RBC, red blood cells; WBC, white blood cells.

#### 3.1.1 Pairwise Comparisons: Dunn's Post Hoc Analysis

**Table 3:** Pairwise comparisons of variables based on disease severity using Dunn's post hoc test.

Parameter	Pairwise comparison	Test statistic (*z*)	Adjusted *p* value
WBC count (×10^6^/L)	Mild‐severe	−28.157	< 0.05
	Mild‐critical	−36.253	< 0.05
	Moderate‐severe	−22.971	< 0.05
	Moderate‐critical	−31.067	< 0.05
RBC count (×10^6^/L)	Critical‐mild	32.602	< 0.05
Lymphocytes count (×10^3^/L)	Critical‐moderate	33.508	< 0.05
	Critical‐mild	58.586	< 0.05
	Severe‐moderate	19.236	< 0.05
	Severe‐mild	44.314	< 0.05
Neutrophil count (×10^3^/L)	Mild‐severe	415.50	< 0.05
BS (mg/dL)	Moderate‐severe	−19.528	< 0.05
CRP (mg/dL)	Mild‐severe	448.0	< 0.05
	Mild‐critical	249.0	< 0.05
	Moderate‐severe	1060.0	< 0.05
	Moderate‐critical	627.50	< 0.05
	Severe‐critical	461.50	< 0.05
IL‐6 (pg/mL)	Mild‐severe	475.0	< 0.05
	Mild‐critical	251.50	< 0.05
	Moderate‐severe	1189.0	< 0.05
	Moderate‐critical	693.50	< 0.05
	Severe‐critical	513.0	< 0.05
ESR (mL/heartbeat)	Mild‐severe	461.50	< 0.05
	Mild‐critical	229.0	< 0.05
	Moderate‐severe	1152.0	< 0.05
	Moderate‐critical	585.0	< 0.05
D‐dimer (ng/mL)	Mild‐severe	443.0	< 0.05
	Moderate‐severe	1118.50	< 0.05
	Moderate‐critical	507.0	< 0.05
Total bilirubin (mg/dL)	Mild‐moderate	461.50	< 0.05
	Mild‐severe	442.50	< 0.05
	Mild‐critical	213.50	< 0.05
AST (U/L)	Moderate‐critical	−26.044	< 0.05
LDH (U/L)	Mild‐moderate	506.50	< 0.05
	Mild‐severe	464.50	< 0.05
	Mild‐critical	225.0	< 0.05
	Moderate‐critical	546.0	< 0.05
Creatinine (mg/dL)	Mild‐severe	−26.554	< 0.05
	Mild‐critical	−45.08	< 0.05
BUN (mg/dL)	Mild‐critical	−36.647	< 0.05
Uric acid (mg/dL)	Mild‐moderate	509.0	< 0.05
	Mild‐severe	494.50	< 0.05
	Mild‐critical	251.0	< 0.05
	Moderate‐severe	1046.0	< 0.05
	Moderate‐critical	610.0	< 0.05
INR (s)	Moderate‐severe	−17.691	< 0.05
PT (s)	Mild‐critical	−28.89	< 0.05
PO_2_ (mm/Hg)	Severe‐mild	30.97	< 0.05
	Severe‐moderate	33.73	< 0.05

The Kruskal–Wallis *H* test performed on key clinical parameters indicated an overall statistically significant difference (*p* < 0.05) among the three independent patient groups (Mild, Moderate, and Severe/Critical) for several variables (as detailed in Table 2). However, the Kruskal–Wallis test does not specify which groups differ from one another (Table 3). To identify the specific location of these differences, we subsequently performed Dunn's post hoc test for pairwise comparisons on all variables that yielded an overall statistically significant result. The *p* values from Dunn's test were adjusted using the Bonferroni correction to control. The complete results of the pairwise comparisons, including the specific group contrasts (e.g., Mild vs. Moderate, Moderate vs. Severe/Critical), the Bonferroni‐adjusted p‐values, and the indication of statistical significance, are presented in Table 3. This table confirms the specific group differences that underpin our conclusions regarding the clinical course and severity of the disease.

Among these findings, WBC counts were significantly different between the mild‐severe, mild‐critical, moderate‐severe, and moderate‐critical groups. Other parameters with overall significance, including RBC count, lymphocyte count, CRP, IL‐6, ESR, and the remaining laboratory findings, are detailed in Table 3 with their respective pairwise comparisons. This table provides a clear overview of the significant differences between severity groups, highlighting how laboratory findings change with increasing COVID‐19 severity.

Furthermore, the present study has effectively identified significant fluctuations in laboratory parameters among individuals diagnosed with COVID‐19, based on the stage of the disease and the hospitalization status of the patients It is worth mentioning that a statistically significant difference in white blood cell count (WBC) was observed only among patients admitted to the CCU (*p* < 0.05), while no significant differences were found in patients in the infectious ward, or those in the ICU. For a more comprehensive understanding of these results, please refer to Table 4.

**Table 4:** Analysis of laboratory parameters and predictors of development and severity, COVID‐19 hospitalized patients, indicated as *N* (mean rank).

Laboratory parameters of hospitalized patients (unit, normal range)	Hospitalizations ward	*N* (mean rank)	*p* value, Mann–Whitney test
WBC count (×10^3^/L, range 4–10)	Infectious ward		0.10
	Yes	39 (62.82)	
	No	72 (52.31)	
	CCU ward		< 0.05
	Yes	16 (71.97)	
	No	95 (53.31)	
	ICU ward		0.095
	Yes	26 (65.21)	
	No	85 (53.18)	
Plt count (×10^3^/L, range 150–450)	Infectious ward		0.148
	Yes	39 (50)	
	No	72 (59.25)	
	CCU ward		0.130
	Yes	16 (67.28)	
	No	95 (54.10)	
	ICU ward		0.577
	Yes	26 (52.92)	
	No	85 (56.94)	
Hb (g/dL)	Infectious ward		0.802
	Yes	39 (54.96)	
	No	72 (56.56)	
	CCU ward		0.740
	Yes	16 (53.53)	
	No	95 (56.42)	
	CCU ward		0.076
	Yes	26 (46.19)	
	No	85 (59)	
Lymphocytes count (×10^3^/L, range 1.1–3.2)	Infectious ward		0.706
	Yes	39 (54.44)	
	No	72 (56.85)	
	CCU ward		0.126
	Yes	16 (44.63)	
	No	95 (57.92)	
	ICU ward		< 0.05
	Yes	26 (37.92)	
	No	85 (61.53)	
Neutrophil count (×10^3^/L, range 1.8–6.3)	Infectious ward		0.089
	Yes	39 (63.05)	
	No	72 (52.18)	
	CCU ward		< 0.05
	Yes	16 (71.38)	
	No	95 (53.41)	
	ICU ward		0.556
	Yes	26 (52.75)	
	No	85 (56.99)	
RP (mg/dL, range 0.0–6.0)	Infectious ward		0.978
	Yes	39 (55.88)	
	No	72 (56.06)	
	CCU ward		0.933
	Yes	16 (56.63)	
	No	95 (55.89)	
	ICU ward		< 0.05
	Yes	26 (79.48)	
	No	85 (48.82)	
IL‐6 (pg/mL)	Infectious ward		< 0.05
	Yes	39 (45.83)	
	No	72 (61.51)	
	CCU ward		0.364
	Yes	16 (62.75)	
	No	95 (54.86)	
	ICU ward		< 0.05
	Yes	26 (85.37)	
	No	85 (47.02)	
D‐Dimer (ng/mL, < 500)	Infectious ward		0.643
	Yes	39 (54.08)	
	No	72 (57.04)	
	CCU ward		< 0.05
	Yes	16 (71.09)	
	No	95 (53.46)	
	ICU ward		< 0.05
	Yes	26 (67.25)	
	No	85 (52.56)	
		Mean ± SD	Wilcoxon signed‐rank test
ESR (mL/heartbeat)	Infectious ward	33.87 ± 29.14	0.232
	CCU ward	51.68 ± 29.65	< 0.05
	ICU ward	48.19 ± 30.23	< 0.05

*Note:* Mann–Whitney test, *p* < 0.05 was considered statistically significant.

### 3.2 Genotyping of the SFTPD SNPs

**Figure 1:**
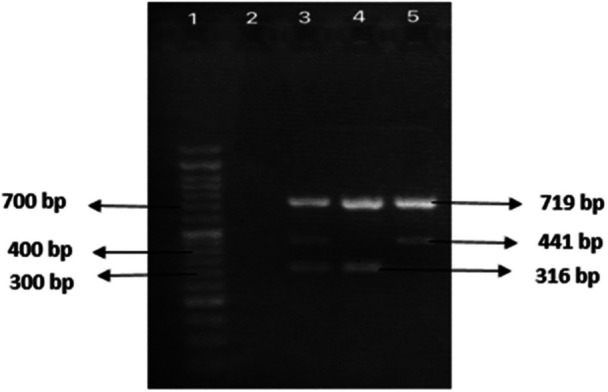
The electrophoresis results obtained from the T‐Arms‐PCR amplification of SFTPD gene SNPs (rs721917, T > C) have been visually shown on a 2% agarose gel. Lane 1: 50‐bp ladder (Cinnagen, Iran); Lane 2: no template control; Lane 3: CT heterozygote; TT homozygous mutant; and Lane 5: CC homozygous reference genotype.

The Tetra‐ARMS‐PCR technique facilitates the differentiation of wild‐type, heterozygous, and homozygous genotypes. The wild‐type genotype generates a 441‐bp PCR product, while the homozygous mutant genotype produces a 316‐bp amplicon. Both alleles yield a 719‐bp control. Heterozygotes display fragments of 441/316‐bp and a 719‐bp control. These variations in product lengths allow for straightforward genotype discrimination through gel electrophoresis. When conducting practical T‐ARMS PCR for SNP detection, it is imperative to consider several factors, including DNA extraction, annealing temperature, PCR cycles, reagent selection, and primer concentrations. Among these considerations, the annealing temperature plays a particularly critical role (see Figure 1).

Table 5 presents the allele and genotype distribution in COVID‐19 patients. The TT genotype T allele exhibited a higher frequency in patients across different severity stages and was associated with disease severity stages (*p* < 0.05). A comparison of the observed and expected counts of genotypic and allelic frequencies of the SFTPD SNPs showed that the TT genotype occurred at a higher‐than‐expected frequency among patients with moderate, severe, and critical forms of COVID‐19 (*p* < 0.05). Regarding gender, the frequencies of the TT genotype were 52.3% and 47.7% among males and females, respectively. The frequencies of the CT genotype were 26.5% and 73.5%, and the frequencies of the CC genotype were 41.7% and 58.3%. The chi‐square test revealed a significant association between the genotype, allele frequencies of the SFTPD T/C gene variant (rs721917 T > C), and disease severity at different stages of the disease (*p* < 0.05). Furthermore, we observed an association between gender and the genotype frequencies or allele distribution of SFTPD (rs721917) among all participants (*p* < 0.05). No significant association was found between the different genotype frequencies and the ages of the participants (*p* > 0.05). The genomic data for the TT, CT, and CC genotypes in this study are available in the NCBI database under the accession numbers OR999130, OR987513, and OR999129. This database provides comprehensive insights into gene characteristics and location, contributing to our comprehension of genetic variations and their impact on health.

**Table 5:** Distribution of genotypic and allelic count (expected count) of the SFTPD SNPs among COVID‐19 patients based on the disease's severity.

SNP		Subjects count (expected count)	Severity count (expected count)				Asymptomatic	*p* value chi‐square test
			Mild	Moderate	Severe	Critical		
SFTPD rs721917 genotypes count (expected count)	TT	65 (65.0)	3 (7.2)	23 (20.2)	24 (17.8)	13 (8.2)	2 (11.6)	< 0.05
	CT	34 (34.0)	7 (3.8)	12 (10.6)	8 (9.3)	3 (4.3)	4 (6)	
	CC	36 (36.0)	5 (4.0)	7 (11.2)	5 (9.9)	1 (4.5)	18 (6.4)	
Total genotypes	135	15	42	37	17	24		
Allel count (expected count)	T	164 (164)	13 (21.1)	58 (59)	56 (52)	29 (23.8)	8 (29.2)	0.05
	C	106 (106)	17 (8.9)	26 (25)	18 (22)	5 (10.2)	40 (18.8)	0.05

*Note:* A significance threshold of chi‐square test, *p* < 0.05 was utilized.

Abbreviations: SFTPD, surfactant protein D; SNP, single‐nucleotide polymorphism.

### 3.3 Genotype Frequencies, Disease Severity, and Survival in COVID‐19 Patients

Table 6 provides an overview of genotype frequencies in various subject groups, categorized by disease severity and hospitalization status. In the hospitalized patient group, the frequencies of TT, CT, and CC genotypes in the SFTPD gene polymorphism (rs721917) were 76.9%, 61.8%, and 27.8%, respectively. These frequencies were compared to those of the outpatient and asymptomatic subjects. These findings indicate a genotype‐frequency‐related risk of hospitalization, particularly in the cardiac care unit (CCU) and ICU wards (*p* < 0.05). Our study revealed that the TT genotype showed higher observed frequencies than expected among ICU and CCU patients, and this difference was statistically significant (*p* < 0.05). In contrast, no significant difference was observed between the observed and expected frequencies in outpatients or patients admitted to the Infectious ward (*p* > 0.05). Additionally, the number of hospitalized patients in the TT genotype group was 3.8 times higher than that of outpatients (*p* < 0.05). However, there was no correlation between the mortality rate and genotype frequency (*p* > 0.05).

**Table 6:** Genotype frequencies in COVID‐19 patients' hospitalization status and mortality.

Variables evaluated	COVID‐19 patients genotype of SFTPD T/C rs721917 T>C count (expected count)			*p* value chi‐square test
	TT	CT	CC	
				0.122
Outpatient	13 (17)	9 (8.1)	8 (4.9)	
Infectious ward	50 (46)	21 (21.9)	10 (13.1)	
CCU ward				
Yes	15 (9.1)	1 (4.3)	0 (2.6)	< 0.05
No	48 (53.9)	29 (25.7)	18 (15.4)	
ICU ward				
Yes	20 (14.8)	5 (7)	1 (4.2)	< 0.05
No	43 (48.2)	25 (23)	17 (13.8)	
Survival				
Yes	55 (57.3)	29 (27.3)	17 (16.4)	0.288
No	8 (5.7)	1 (2.7)	1 (1.6)	

*Note:* Statistical significance was considered at the chi‐square test, *p* < 0.05.

Abbreviations: CI, confidence interval; Covid‐19, coronavirus disease 2019; ICU, intensive care unit; OR, odds ratio; SFTPD, surfactant protein D.

This study establishes a significant association between genotype frequency, certain characteristics, and clinical findings in all subjects. For example, gender, smoking status, and comorbid conditions such as diabetes were compared among different disease stages (*p* < 0.05). Although genotype frequencies did not show a clear association with the history of cancer and hypertension in each patient group separately (*p* > 0.05).

## 4 Discussion

This research underscores the significant influence of host genetic variations on an individual's susceptibility to severe symptoms of COVID‐19 and other infectious diseases. Our study identified that the SFTPD gene rs721917 T > C polymorphism, particularly the T allele and TT genotypes, is associated with an increased risk of SARS‐CoV‐2 infection and heightened disease severity; these genotypes were observed to be more prevalent in individuals exhibiting severe disease stages compared to those presenting with mild symptoms, as well as in asymptomatic or presymptomatic patients. An understanding of these genetic underpinnings is essential for the development of targeted therapeutic strategies. COVID‐19 manifests with a range of clinical symptoms and leads to abnormal laboratory parameters, making it imperative to identify all clinical indicators of the disease to facilitate early diagnosis and the implementation of preventive measures [31, 32].

The analysis of hematological and physiological parameters across severity groups revealed patterns consistent with previous studies, thereby confirming established associations with disease progression. Consequently, these results validate earlier observations rather than present novel findings. The main contribution of the present study lies in the identified association between the SP‐D rs721917 polymorphism and COVID‐19 outcomes.

Previous research has highlighted that the timely identification of primary symptoms of COVID‐19, such as fever, cough, fatigue, and dyspnea, is crucial in mitigating the transmission of the virus. Additionally, comorbidities including hypertension and diabetes have been shown to exacerbate the clinical course in individuals infected with COVID‐19 [33, 34]. Furthermore our study established a significant association between disease severity and specific symptoms, namely fever, dry cough, myalgia and dyspnea, as well as underlying conditions such as diabetes and hypertension, particularly in severe and critical cases as opposed to mild cases (*p* < 0.05).

Furthermore, recent research has emphasized a correlation between COVID‐19 patients and electrolyte imbalances (specifically low sodium, potassium, and calcium levels), renal impairment, and coagulation abnormalities [35, 36, 37]. We demonstrated fluctuations in these parameters and their relationship to disease stage. Recent meta‐analyses have corroborated earlier findings by identifying elevated levels of inflammatory markers, ESR, C‐reactive protein (CRP), procalcitonin (PCT), and ferritin, alongside increased white blood cell (WBC) counts and decreased lymphocyte counts in COVID‐19 cases. These indicators have consistently been associated with disease severity [38].

This study identified a correlation between the gene variant rs721917 and clinical parameters in both severe and non‐severe cases of COVID‐19. Elevated levels of inflammatory markers and liver and kidney enzymes were observed in patients in the ICU, accompanied by a decreased lymphocyte count. These factors were associated with the progression and prognosis of severe COVID‐19. The SFTPD gene rs721917 T > C polymorphism, specifically the T allele and TT genotype, was found to increase the risk of SARS‐CoV‐2 infection and was more prevalent in patients experiencing severe stages of the disease compared to those with mild symptoms, as well as asymptomatic or presymptomatic individuals. Furthermore, it was associated with hospitalization status and was observed more frequently in patients hospitalized in the ICU and CCU wards.

SP‐D contributes to innate immunity by binding to viral surface glycoproteins, such as influenza hemagglutinin, which facilitates viral clearance. It also modulates immune responses through interactions with macrophages and the activation of p38 MAPK [39]. In cases of lung injury, SP‐D is primarily synthesized and secreted into the alveolar space by alveolar type II epithelial cells. The translocation of SP‐D into the bloodstream typically occurs in the context of damage to the epithelial or endothelial components of the alveolar‐capillary barrier. Its presence in the circulation is widely recognized as a biomarker of alveolar epithelial injury and increased alveolar‐capillary permeability, rather than a direct contributor to disease pathology [40, 41]. Accordingly, in cases of lung injury, elevated serum SP‐D levels have been used to assess alveolar damage and inflammation in conditions such as ARDS [39]. Recent findings suggest that in COVID‐19 patients, increased serum SP‐D levels are more strongly associated with mortality than with clinical severity, indicating that SP‐D primarily reflects alveolar injury rather than playing a direct pathogenic role [27]. Recent findings indicate that specific genetic variants of human SP‐A have distinct roles in modulating lung injury caused by SARS‐CoV‐2, affecting both viral infectivity and the host's inflammatory responses. Additionally, evidence of surfactant protein dysregulation in COVID‐19 suggests that personalized surfactant‐based therapies could be advantageous for high‐risk patients [42]. While direct clinical and epidemiological evidence supporting the role of SP‐A and SP‐D in respiratory viral infections remains limited, partly due to the redundancy of innate immune mechanisms, insights have emerged through genetic and observational studies. Complete inherited deficiencies of SP‐A or SP‐D have not been documented; however, certain polymorphisms, such as Thr11 and Ala160 in SP‐D, have been associated with decreased severity of RSV infection, potentially due to SP‐D's binding to the RSV G protein. Additionally, SP‐D has been shown to inhibit HIV infectivity by interacting with gp120 and impeding viral entry into CD4^+^ cells, although this activity is less pronounced than that against the influenza A virus [43]. Recent research findings indicate that the SP‐D, which is derived from the SFTPD gene, holds potential as a biomarker for predicting severe COVID‐19 pneumonia. Alterations in the levels of SP‐D in blood serum have been observed in correlation with the severity of COVID‐19 and have demonstrated possible associations with genetic variations in the SFTPD gene [27]. Studies have highlighted the relationship between this polymorphism and respiratory diseases, suggesting a potential role in predisposing individuals to severe outcomes related to COVID‐19 [44]. Ghaniam et al. reported that specific variations in the SP‐D gene, particularly the rs3088308 and rs721917 T allele, have been associated with elevated levels of SP‐D in the serum of patients with COPD. The rs721917 CT genotype and T allele have been found to exhibit a higher prevalence among COPD patients. Moreover, smoking status has been shown to have a stronger association with the TT and CT genotypes compared to the CC genotype [45].

This study posits that SP‐D may serve as a significant biomarker for assessing the severity of COVID‐19. In the current investigation, the prevalence of the TT genotype was found to be correlated with increased severity of COVID‐19 and was more frequently observed in smokers. Moreover, this genotype exhibited a relationship with smoking status in comparison to the CC genotype. Additional research has identified a strong association between elevated levels of SP‐D and angiopoietin‐2 (Ang‐2) with the severity of COVID‐19, thereby suggesting their potential utility in prognosticating and differentiating patient outcomes [29]. Our findings indicate a potential association between the SP‐D SNP rs721917 and the severity of COVID‐19, particularly when compared to asymptomatic or presymptomatic individuals. Genetic variants associated with human surfactant proteins have been shown to increase susceptibility to respiratory disorders, indicating their role in both chronic and acute respiratory diseases [37]. Recent case–control studies have elucidated the impact of specific genetic variants across various genes on the severity and susceptibility to COVID‐19 [46, 47]. The SP‐D SNP rs721917 has been linked to a variety of diseases, underscoring its involvement in disease onset and its association with various polymorphisms. Rizvi et al. demonstrated an association between SP‐D polymorphisms (SNP rs2243639 T > C, Thr180Ala), the rs3088308 A > T, the Ser290Thr variant, and the rs721917 genotype T/T with recurrent acute stomatitis (RAS) in the Pakistani population.

The T allele demonstrated a correlation with RAS, particularly among females, while no significant correlation was identified with BMI range or hemoglobin levels [48]. In our study, we noted a correlation between gender and genotype frequency, with the TT genotype being more prevalent among males. However, no association was found between BMI, hemoglobin levels, and genotype frequencies across all participants. Lahti et al. reported that the SP‐D Met11Thr polymorphism contributes to individual susceptibility to severe RSV bronchiolitis in infants, with the Met/Met genotype indicating a heightened vulnerability to severe infection [19]. Conversely, Fakih et al. did not establish any correlation between the SP‐D gene SNP rs721917 (Met11Thr) and COPD or asthma within the Lebanese population, thus confirming the absence of an association with these respiratory disorders [11]. Similarly, Petersen et al. found no significant association between SFTPD genotypes (rs2243639 C/T, rs3088308 A/T, and rs721917 A/G) and clinical parameters in Danish adolescents and young adults diagnosed with mild to moderate asthma. Furthermore, no association was observed between serum SP‐D levels and SFTPD genotypes concerning the clinical parameters of asthma [49].

However, no correlation was observed between genotype prevalence and mortality rate in the present study. Lin et al. reported an association between the rs2243639 (G/A Ala160Thr) polymorphism and Crohn's disease; however, no association was identified between rs721917 (C/T Met11Thr) and inflammatory bowel disease. The comparable allele frequencies suggest that there may be a limited influence on protein function or incomplete genetic information transmission [50]. Hsieh et al. revealed that the SP‐D 92T (amino acid residue 16, methionine) genotype, associated with the C92T (rs721917) exonic variation, increases susceptibility to pulmonary tuberculosis. Individuals possessing this genotype demonstrate weaker binding to *Mycobacterium bovis* BCG and reduced effectiveness in inhibiting intracellular growth compared to those with the 92T (amino acid residue 16, threonine) variant, thereby emphasizing the role of SP‐D in pulmonary immunity against mycobacterial infections [51]. Similarly, Liu et al. conducted a study highlighting the significant impact of the SP‐D polymorphism rs721917 CC genotype in predicting the incidence of acute kidney injury (AKI) and elevated mortality rates in septic patients [52]. These findings suggest a potential association between these genotypes and a slightly increased risk of disease severity, thereby providing valuable insights into the role of genetic factors in COVID‐19 susceptibility and severity. The specific variant prevalent in severe cases in southern Iran may have clinical implications for the management of COVID‐19. The present study underscores the significance of genetic variations in influencing disease severity and highlights the crucial role of SP‐D in immune response and host defense. Nevertheless, it is essential to acknowledge the limitations of this study; it is imperative to conduct more extensive investigations across diverse populations. An examination of the rs721917 polymorphism during the emergence of the Omicron variant (B.1.1.529) in Iran has been conducted. It is important to note that this study does not include CT scan data from patients. Although the potential role of the SP‐D rs721917 (Met31Thr) polymorphism in the development of long COVID symptoms has not been assessed in the present study, future investigations are warranted to determine whether this variant contributes to post‐acute sequelae of SARS‐CoV‐2 infection. Furthermore, future studies should focus on investigating the relationship between serum levels of SP‐D and the frequencies of various genotypes in conjunction with other SARS‐CoV‐2 variants among COVID‐19 patients. Nonetheless, our findings underscore the necessity for further research into other polymorphisms and their impact on the global prevalence and mortality rates of COVID‐19.

The sample size may limit the statistical power to detect significant associations, particularly for variants with small effect sizes. Misclassification bias, arising from variability in COVID‐19 testing sensitivity, could also impact the accuracy of our findings. Population stratification, if not adequately controlled, may lead to spurious associations. Furthermore, the absence of universal testing and comprehensive individual exposure information presents challenges in accurately identifying control groups and assessing genetic susceptibility. Key confounders, including comorbidities, age, socioeconomic status, environmental exposures, and behavioral factors, must be carefully adjusted to isolate the genetic effects on COVID‐19 outcomes. Genetic heterogeneity within the population further complicates the analysis, necessitating careful consideration to ensure robust and reliable conclusions.

Based on the current findings, it is recommended that future research concentrate on investigating the combined effects of multiple SNPs on the severity of COVID‐19 to achieve a more comprehensive understanding of genetic susceptibility. Specifically, studies could examine the interactions between various SNPs within the SP‐D gene and other pertinent genes involved in immune response and lung function. This approach may facilitate the identification of genetic profiles that confer a higher or lower risk of severe COVID‐19 outcomes. Furthermore, conducting longitudinal studies to assess how these genetic variations influence disease progression and response to treatment over time would enhance our understanding of the role of genetics in COVID‐19. By delineating these specific hypotheses, we aim to expand the scope of our research and establish a more focused agenda for future investigations.

## 5 Conclusion

This study identified a clear and positive correlation between variations in the SFTPD gene, particularly the rs721917 T/C polymorphism, and outcomes related to COVID‐19. Homozygosity of the SFTPD T > C mutation was associated with increased severity of COVID‐19. Despite the proliferation of research examining genetic factors influencing COVID‐19 outcomes, there remains a significant gap in the understanding of the specific role of SP‐D in the immune response to SARS‐CoV‐2 infection. This study aimed to address this gap by providing a comprehensive analysis of how variations in the SP‐D gene impacted disease progression, thereby offering new insights into the genetic factors that dictate the severity of COVID‐19. By emphasizing the critical role of genetic variations in disease outcomes, this study carries important implications for public health and personalized medicine. Specifically, the integration of SNP‐based genetic screening into clinical practice could facilitate the early identification of individuals predisposed to severe COVID‐19. Such predictive capabilities would enable tailored therapeutic interventions and targeted preventive strategies, ultimately enhancing patient outcomes and optimizing the allocation of healthcare resources. Future research should concentrate on validating these genetic markers across diverse populations and exploring the feasibility of implementing genetic screening protocols in routine clinical settings to improve the precision of COVID‐19 management. Our findings suggest that genetic profiling of SP‐D polymorphisms could be instrumental in identifying individuals at higher risk, thereby informing personalized therapeutic strategies and improving clinical outcomes. However, it is essential to recognize that factors beyond genetics may also influence the interplay between underlying diseases, COVID‐19 severity, and susceptibility to the virus. Consequently, the complex pathophysiology of severe COVID‐19 cannot be fully elucidated by examining genetic risk factors alone, underscoring the multifaceted nature of the disease and the necessity for further comprehensive investigation.

## Author Contributions

**Nadia Nasirzadeh Kolsari:** writing – original draft preparation, conceptualization, methodology, investigation, software, formal analysis, resource, visualization. **Azarakhsh Azaran:** writing – review and editing, validation, data curation, supervision. **Roya Pirmoradi:** investigation, validation, formal analysis. **Maryam Moradi:** formal analysis, data curation, validation. **Roohangiz Nashibi:** investigation, data curation, validation. **Shahram Jalilian:** writing – review and editing, conceptualization, validation, data curation, supervision, project administration, funding acquisition. All authors have read and approved the final version of the manuscript.

## Ethics Statement

This study, which included human participants, complied with the ethical standards established in the 1964 Helsinki Declaration and received approval from the Ethics Committee of Jundishapur University of Medical Sciences, Ahvaz, Iran (IR.AJUMS.REC.1401.503). Written informed consent was obtained from all participants.

## Conflicts of Interest

The authors declare no conflicts of interest.

## Supporting information

Supporting File 1

Supporting File 2

Supporting File 3

## Data Availability

Data will be made available on request.
