# Supplementary material for: The Impact of Surfactant Protein‐D Gene Polymorphism on COVID‐19 Clinical Outcomes
Source: Immun Inflamm Dis. 2026 May 15;14(5):e70410. doi: 10.1002/iid3.70410 (PMC13178794; doi:10.1002/iid3.70410)
Supplement: Supplementary file 1 — Supporting File 1 [file IID3-14-e70410-s002.doc]

STROBE Statement—Checklist of items that should be included in reports of ***case-control studies***

|  | Item No | Recommendation |  | Page NO. | Relevant text from manuscript |
| --- | --- | --- | --- | --- | --- |
| **Title and abstract** | 1 | (*a*) Indicate the study’s design with a commonly used term in the title or the abstract |  | 1 | Lines 1,2 |
|  |  | (*b*) Provide in the abstract an informative and balanced summary of what was done and what was found |  | 1,2 | Lines 25-50 |
| Introduction | | |  |  |  |
| Background/rationale | 2 | Explain the scientific background and rationale for the investigation being reported |  | 2-4 | Lines 52-133 |
| Objectives | 3 | State specific objectives, including any prespecified hypotheses |  | 4 | Lines 128-133 |
| Methods | | |  |  |  |
| Study design | 4 | Present key elements of study design early in the paper |  | 4-7 | Lines 134-242 |
| Setting | 5 | Describe the setting, locations, and relevant dates, including periods of recruitment, exposure, follow-up, and data collection |  | 4-6 | Lines 134-177 |
| Participants | 6 | (*a*) Give the eligibility criteria, and the sources and methods of case ascertainment and control selection. Give the rationale for the choice of cases and controls |  | 4,5 | Lines 139-167 |
|  |  | (*b*) For matched studies, give matching criteria and the number of controls per case |  | 4-6 | Lines 143-177 |
| Variables | 7 | Clearly define all outcomes, exposures, predictors, potential confounders, and effect modifiers. Give diagnostic criteria, if applicable |  | 5,6 | Lines 149-177 |
| Data sources/ measurement | 8* | For each variable of interest, give sources of data and details of methods of assessment (measurement). Describe comparability of assessment methods if there is more than one group |  | 4-7 | Lines 139-242 |
| Bias | 9 | Describe any efforts to address potential sources of bias |  | 17 | Lines 481-490 |
| Study size | 10 | Explain how the study size was arrived at |  | 7 | Lines 230,231 |
| Quantitative variables | 11 | Explain how quantitative variables were handled in the analyses. If applicable, describe which groupings were chosen and why |  | 5,7,8 | Lines 150, 233-242 (Table1 262-266) |
| Statistical methods | 12 | (*a*) Describe all statistical methods, including those used to control for confounding |  | 7 | Lines 229-242 |
|  |  | (*b*) Describe any methods used to examine subgroups and interactions |  | 7,9,10,11,12,13 | Lines 229-242,266,280,291,329,353 |
|  |  | (*c*) Explain how missing data were addressed |  | N/A | All required data were available |
|  |  | (*d*) If applicable, explain how matching of cases and controls was addressed |  | 5,7 | Lines 146-148,231-242 |
|  |  | (*e*) Describe any sensitivity analyses |  | N/A | Basic case-control design – sensitivity analysis was not conducted |
| Results | | |  |  |  |
| Participants | 13* | (a) Report numbers of individuals at each stage of study—eg numbers potentially eligible, examined for eligibility, confirmed eligible, included in the study, completing follow-up, and analysed |  | 4,5,8 | Lines 138,143,(Table1 263 The number of patients in each groups) |
|  |  | (b) Give reasons for non-participation at each stage |  | N/A | Lines This data were not systematically recorded due to the study's retrospective design. |
|  |  | (c) Consider use of a flow diagram |  | N/A | using pre-existing datasets without screening steps. |
| Descriptive data | 14* | (a) Give characteristics of study participants (eg demographic, clinical, social) and information on exposures and potential confounders |  | 8 | Lines 263-266 |
|  |  | (b) Indicate number of participants with missing data for each variable of interest |  | N/A | This data were not systematically recorded due to the study's retrospective design. |
| Outcome data | 15* | Report numbers in each exposure category, or summary measures of exposure |  | 4,5,8 | Lines 138,143,(Table1 263 The number of patients in each groups) |
| Main results | 16 | (*a*) Give unadjusted estimates and, if applicable, confounder-adjusted estimates and their precision (eg, 95% confidence interval). Make clear which confounders were adjusted for and why they were included |  | 7,12,13 | Lines 237-242, 331-343,(Table5 353) |
|  |  | (*b*) Report category boundaries when continuous variables were categorized |  | 5 | Lines 149-167 |
|  |  | (*c*) If relevant, consider translating estimates of relative risk into absolute risk for a meaningful time period |  | N/A | Insufficient data to compute absolute risk |

| Other analyses | 17 | Report other analyses done—eg analyses of subgroups and interactions, and sensitivity analyses 8,9 Lines (Table1 263-266), Sensitivity analyses not conducted |  |  |
| --- | --- | --- | --- | --- |
| Discussion | | |  |  |
| Key results | 18 | Summarise key results with reference to study objectives 14 Lines 355-367 |  |  |
| Limitations | 19 | Discuss limitations of the study, taking into account sources of potential bias or imprecision. Discuss both direction and magnitude of any potential bias 16,17 Lines 468-490 |  |  |
| Interpretation | 20 | Give a cautious overall interpretation of results considering objectives, limitations, multiplicity of analyses, results from similar studies, and other relevant evidence 16,17,18 Lines 462-490,501-525 |  |  |
| Generalisability | 21 | Discuss the generalisability (external validity) of the study results 16,17 Lines 469-500 |  |  |
| Other information | | |  |  |
| Funding | 22 | Give the source of funding and the role of the funders for the present study and, if applicable, for the original study on which the present article is based 18 Lines 547-548 |  |  |
